# Synthesis, Spectral Analysis, *In Vitro* Microbiological Evaluation, and Molecular Docking Studies of Some Novel 1-(1-Aryl-1*H*-tetrazol-5-yl)-2-(piperidin-1-yl)ethanone Derivatives

**DOI:** 10.1155/2014/120173

**Published:** 2014-05-06

**Authors:** Thangasamy Elavarasan, Durairaj Peter Bhakiaraj, Mannathusamy Gopalakrishnan

**Affiliations:** Synthetic Organic Chemistry Laboratory, Department of Chemistry, Annamalai University, Annamalai Nagar, Tamil Nadu 608 002, India

## Abstract

A new series of novel heterocyclic compounds containing both tetrazoles and piperidine nuclei together, namely, 1-(1-aryl-1*H*-tetrazol-5-yl)-2-(piperidin-1-yl)ethanone (**22**–**28**), were synthesized by the treatment of the respective 2-chloro-1-(1-aryl-1*H*-tetrazol-5-yl)ethanone (**15**–**21**) with piperidine in acetonitrile for 6 h. A series of novel tetrazole substituted piperidine derivatives were synthesized and evaluated for their antimicrobial activity using serial dilution method. The structures of the synthesized compounds were characterized by IR, ^1^H NMR, ^13^C NMR, mass spectral data, and elemental analysis. Evaluation of antimicrobial activity shows that several compounds exhibit good activity when compared with the reference drug candidates and thus could be promising new lead molecules.

## 1. Introduction

**Figure 1:**
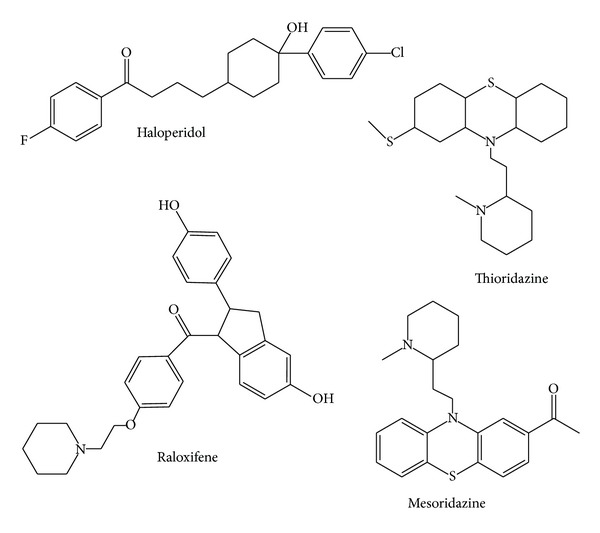
Biologically active piperidine containing drugs.

A wide variety of heterocyclic systems have been explored for developing pharmaceutically important molecules. Heterocycles form by far the largest of the classical divisions of organic chemistry [1]. Generally N-based heterocycles have been the object of considerable focus because N-containing heterocycles are structural components of many bioactive natural products such as vitamins, hormones, antibiotics, alkaloids, glycosides, and many more compounds which are of significance for human and animal health [2]. Many natural drugs such as quinine, papaverine, emetine, theophylline, atropine, codeine, morphine, and reserpine are N-containing heterocycles [3]. Therefore, N-containing heterocycles are especially considered ‘‘privileged” structures for the synthesis and development of new drugs [4, 5]. Tetrazoles and their derivatives have been reported as antibacterial [6], antiviral [7], herbicidal [8], anti-inflammatory [9], antitumor [10], analgesic [11], and antiproliferative [12] agents. Moreover, tetrazoles are representing an important structural motif in medicinal chemistry. The piperidine ring is a ubiquitous structural feature in many alkaloid natural products and drug candidates [13]. Watson et al. have asserted that, during a recent ten-year period, thousands of piperidine compounds have been mentioned in clinical and preclinical studies [14]. Heterocyclic compounds carrying piperidine skeleton are attractive targets of organic synthesis owing to their pharmacological activity and their wide occurrence in nature. Piperidines and their derivatives have attracted much attention from the scientific community since they represent the core unit of a wide range of alkaloids and biologically active compounds [15–17]. Piperidine nucleus is an important core of many drug molecules. Piperidine and its analogues are reported in literature for varied pharmacological activities like antihistamines and antibacterial [18], AChE inhibitors [19], and antitubercular agents [20]. It is known that clinically useful drugs such as miconazole, bifonazole, clotrimazole, and oxiconazole having an imidazole moiety exhibit strong antimicrobial activity (Figure 1). In view of these observations, it was thought worthwhile to synthesize novel 1-(1-aryl-1*H*-tetrazol-5-yl)-2-(piperidin-1-yl)ethanone derivatives (**22–28**).

## 2. Results and Discussion

### 2.1. Chemistry

**Scheme 1:**
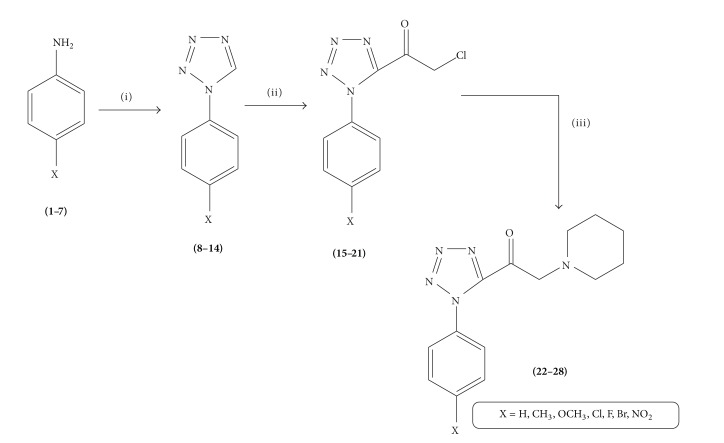
Scheme for the synthesis of 1-(1-aryl-1*H*-tetrazol-5-yl)-2-(piperidin-1-yl)ethanone derivatives (**22–28**). Reagents and conditions: (i) triethylorthoformate, NaN_3_, CH_3_COOH, reflux; (ii) chloroacetyl chloride, THF, pyridine, reflux; (iii) piperidine, CH_3_CN, TEA, RT.

The new 1-(1-aryl-1*H*-tetrazol-5-yl)-2-(piperidin-1-yl)ethanone derivatives (**22–28**) were synthesized according to Scheme 1. Reaction of aryl aniline with sodium azide and triethylorthoformate in acetic acid resulted in the formation of tetrazole compounds (**8–14**). Compounds (**8–14**) on further reaction with chloroacetyl chloride resulted in the formation of 2-chloro-1-(1-aryl-1*H*-tetrazol-5-yl)ethanone (**15–21**). Compounds (**15–21**) further react with piperidine in acetonitrile to get novel 1-(1-aryl-1*H*-tetrazol-5-yl)-2-(piperidin-1-yl)ethanone. All the synthesized compounds were characterized using IR, ^1^H NMR, ^13^C NMR, elemental analysis, and mass spectral studies.

In order to assign the ring proton and carbon signals, compound** 22** has been chosen as representative compound. The ^1^H NMR spectrum of compound** 22** shows a sharp singlet signal at 1.37 ppm with two protons being integral; this is assignable to methylene protons of piperidine. A sharp singlet signal shows 1.51 and 2.45 ppm for their corresponding two methylene protons of piperidine. A sharp singlet appears at 3.15 ppm which supports the presence of methylene proton in ethanone moiety. An aromatic proton appears as a multiplet in the region of 7.07–7.52 ppm. Compound** 22** was confirmed by the presence of carbon signals at 23.36, 25.48, and 53.73 ppm which are assigned to piperidine carbon moiety. An aromatic carbon signal appears in the region of 119.62–128.95 ppm. The phenyl ring ipso carbon and tetrazole ipso carbon appeared at 137.43 and 150.19 ppm, respectively. Carbonyl carbon signal is shown at 172.79 ppm, respectively. The IR spectra of compound** 22 **showed sharp bands appearing at 1651 due to C=O group.

### 2.2. Antibacterial Activity

The* in vitro* antibacterial activity of newly synthesized compounds** 22–28** was determined by serial dilution method (Table 1). All the synthesized compounds** 22–28** were assessed to elicit their antibacterial activity* in vitro* against* Staphylococcus aureus*,* Vibrio cholerae*,* Escherichia coli*,* Salmonella Typhi*, and* Klebsiella pneumoniae*. The antibacterial potency of the synthesized compounds was compared with Ciprofloxacin and their minimum inhibitory concentration (MIC) by serial dilution method; the values were summarized in Table 2. Close surveys of the MIC values indicate that all the compounds exhibited a varied range (6.25–200 *μ*g/mL) of antibacterial activity against all the tested bacterial strains. The MIC values of compounds** 25**,** 27**, and** 28** showed maximum inhibition activity (6.25 µg/mL) against* E. coli.* Among the various substituted compounds, compound** 23** against* S. aureus *and* S. Typhi*, compound** 25** against* K. pneumoniae*, and compound** 23** against* V. cholerae* did not show any activity even at maximum concentration (200 µg/mL). Electron withdrawing substituents like chloro-, fluoro-, and nitro substituted compounds** 25**,** 27,** and** 28** exerted excellent antibacterial activities. Fluorination increases the lipophilicity due to strong electron withdrawing capability of fluorine. Moreover, fluorine substitution was commonly used in contemporary medicinal chemistry to improve metabolic stability, bioavailability, and protein ligand interactions.

### 2.3. Antifungal Activity

In order to extend the antimicrobial evaluation, the antifungal screening revealed that the synthesized compounds (**22–28**) showed good inhibition against various tested fungal strains, namely,* Aspergillus flavus*,* Aspergillus niger*,* Candida albicans*,* Mucor*,* Candida 6*, and* Rhizopus*. Here, Fluconazole was used as standard drug. The result indicated that, among the tested compounds,** 28 **showed maximum inhibition activity (6.25 µg/mL) against* C. albicans.* Among the various substituted compounds, compound** 23** against* A. flavus*, compound** 24** against* Mucor*, and compound** 25** against* Rhizopus *did not show any activity even at maximum concentration (200 µg/mL).

However, the introduction of halogens functionality at* para* position of phenyl groups in compound** 22** registered moderate inhibition potency against all the tested fungal organisms with MIC ranging from 6.25 to 100 µg/mL. Instead of halogens, the nitro substituted compound** 28** shows maximum antifungal potency against* C. albicans*. A modification of* para* proton by chloro, fluoro, and nitro groups in compound** 22** (compounds** 25**,** 27,** and** 28**) shows moderate activity against the entire tested fungal strains but registered high inhibition against* C. albicans *(6.25–25 µg/mL). Results of antifungal studies have been presented in Table 3.

### 2.4. Molecular Docking Studies

**Figure 2:**
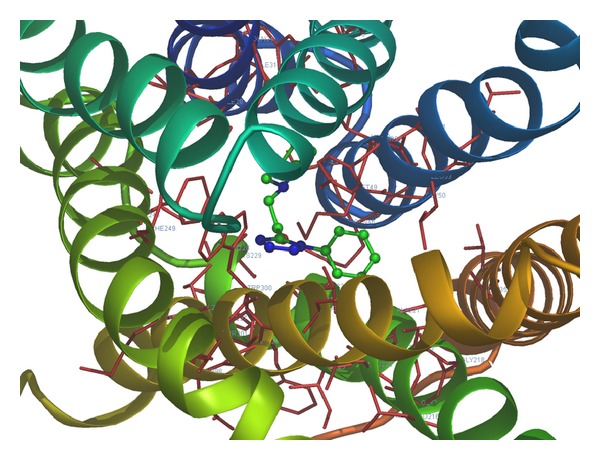
Docked ligand molecule** 22** with the secondary structure of* multidrug transporter EmrD* in solid and ribbon model.

**Figure 3:**
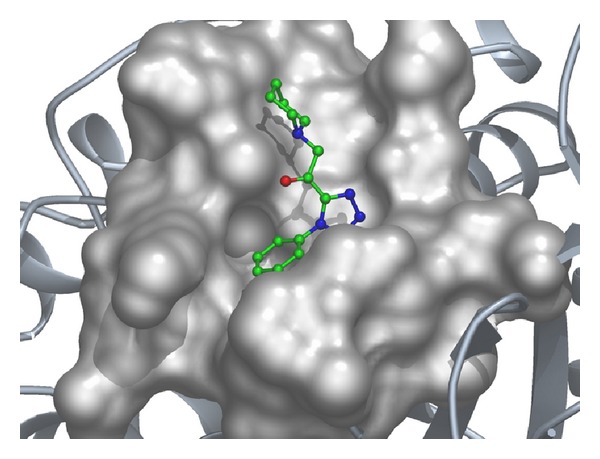
The surface cavity with target molecule** 22** at the active pocket of the protein.

**Figure 4:**
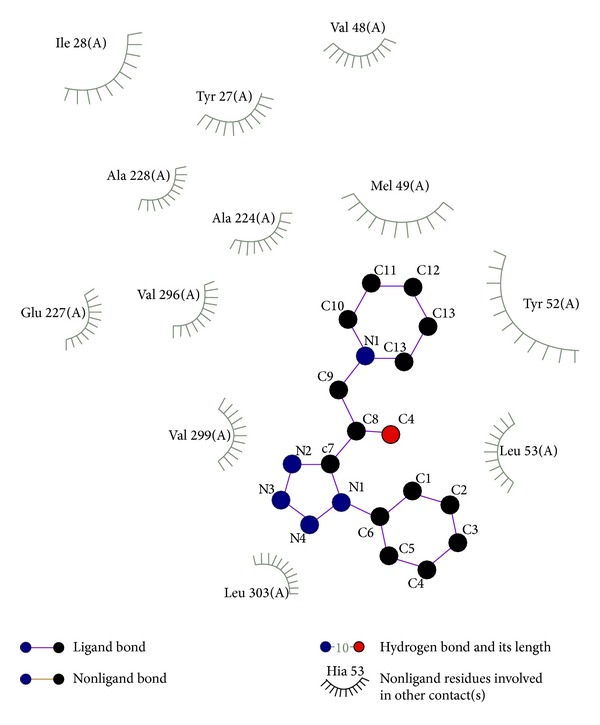
2D plot of hydrogen bond forming amino acids with target ligand for compound** 22**.

**Figure 5:**
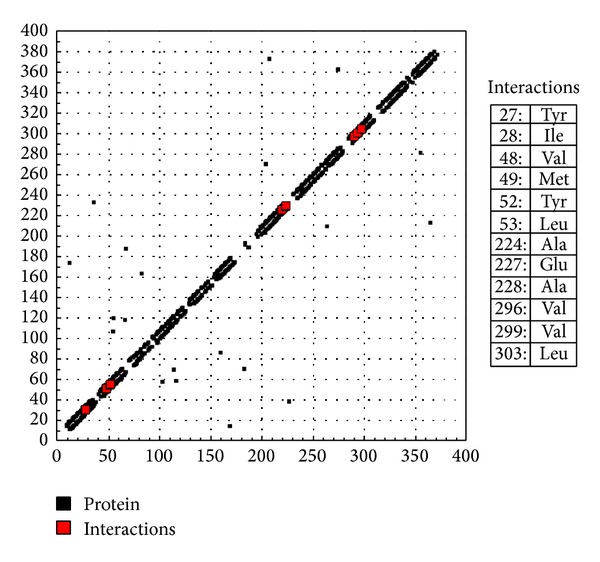
HB plot of interacted residues in protein with compound** 22**.

Considering the well-obtained* in vitro* results, it was thought worthy to perform molecular docking studies for all newly synthesized compounds. Molecular docking study is a well-established technique to determine the interaction of two molecules and find the best orientation of ligand that would form a complex with overall minimum energy. All the newly synthesized compounds** (22–28)** were docked with* multidrug transporter EmrD* of* E. coli* at ten different orientations. The protein structure file (PDB ID:** 2GFP**) taken from PDB (http://www.rcsb.org/pdb/) and the ligands molecules were drawn and analysed using ChemDraw Ultra 8.0.** 3D** coordinates were prepared using DOCK server. The acting force of this binding mode is mainly hydrogen bonding, electrostatic forces, van der Waals forces, and hydrophobic interaction due to nonpolar residue interaction and water structure effect alteration (Aridoss et al. [19]). Based on the* in vitro* antimicrobial studies, it is worthwhile to do* in silico *studies; it supports the* in vitro* activity. The best orientations of hydrogen bonds and hydrophobic interaction of docked molecules are given in Table 4.* In silico* studies revealed that all the synthesized molecules showed good binding energy toward the target protein ranging from −9.57 to −7.68 kcal/mol. The docking results revealed that compounds** 25, 27, **and** 28** showing minimum binding energies −9.57, −8.68, and −9.37 kcal/mol due to dipole-dipole and hydrogen bond interaction with amino acids of targeted protein. Docked ligand molecule** 22** with the secondary structure of* multidrug transporter EmrD* in solid and ribbon model is depicted in Figure 2. The surface cavity with target molecule** 22** at the active pocket of the protein structure is depicted in Figure 3. 2D plot of hydrogen bond forming amino acids with target ligand and HB plot of interacted residues in protein of* E. coli* with compound** 22 **are depicted in Figures 4 and 5, respectively.

**Figure 6:**
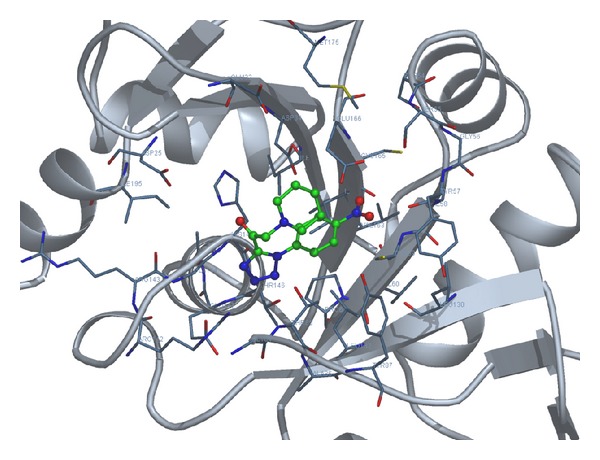
Docked ligand molecule** 28** with the secondary structure of* dihydrofolate reductase* in solid and ribbon model.

**Figure 7:**
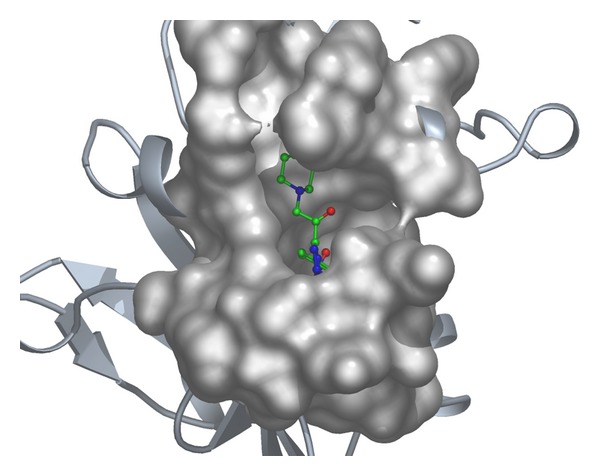
The surface cavity with target molecule** 28** at the active pocket of the protein.

**Figure 8:**
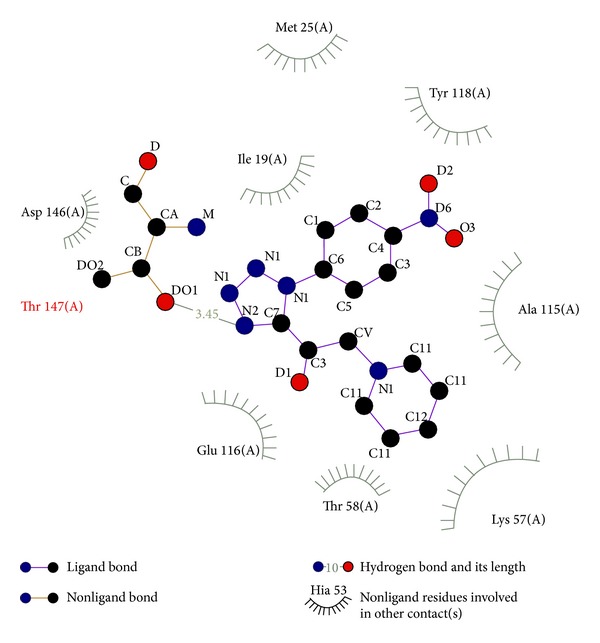
2D plot of hydrogen bond forming amino acids with target ligand for compound** 28**.

**Figure 9:**
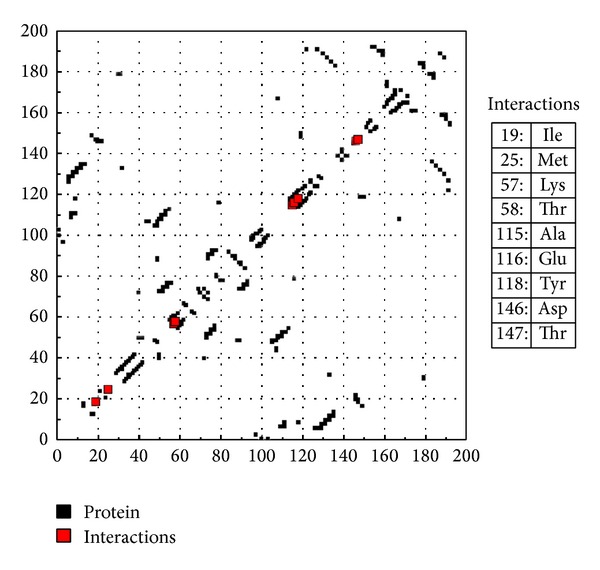
HB plot of interacted residues in protein with compound** 28**.

The* in vitro* antifungal MIC values are correlated well with binding energies obtained through molecular docking with* dihydrofolate reductase* (PDB** 1AI9**) of* C. albicans. *Docked ligand molecule** 28** with the secondary protein structure of* dihydrofolate reductase* in solid and ribbon model is depicted in Figure 6. The minimum fungal inhibition potency* C. albicans* of compounds** 25** (25 µg/mL),** 26** (6.25 µg/mL), and** 28** (12.5 µg/mL) showed excellent docking energies. Its total docking energies are −8.91, − 8.70, − 9.27 kcal/mol, respectively, shown in Table 5. From the comparative analysis, compounds** 27** and** 28** showed minimum docking energy with targeted protein and the* in vitro* studies also support that compounds** 27** and** 28** have become active against all the tested microorganisms. The abovementioned compounds utilize their amino head group to interact with the crucial amino acid residues such as Glu 116, Thr 58, Lys 57, Ala 115, and Asp 146 through the hydrogen bonds. The active binding sites THR 147 were occupied with target ligand molecules at active site of the protein. The surface cavity with target molecule** 13** at the active pocket of the protein structure is depicted in Figure 7. 2D plot of hydrogen bond forming amino acids with target ligand and HB plot of interacted residues in protein of* C. albicans* with compound** 28 **are depicted in Figures 8 and 9, respectively. So, we suggested compound** 28** nitro substituted phenyl group to exhibit better bacterial and fungal inhibition. Therefore, it is pleasing to state that the docking studies have widened the scope of developing a new class of antimicrobial agents.

## 3. Conclusions

In conclusion, a series of novel 1-(1-aryl-1*H*-tetrazol-5-yl)-2-(piperidin-1-yl)ethanone derivatives were synthesized in good yields and their structures were characterized by their IR, ^1^H NMR, ^13^C NMR, and mass spectral data. The synthesized compounds showed a wide range of potentially promising antibacterial and antifungal activities. Compounds** 25**,** 26,** and** 28 **showed significant microbial activity against the tested bacterial and fungal strains. The docking study reveals that hydrophobic interactions played a major role in ligand receptor interactions.

## 4. Experimental

### 4.1. Chemistry

All the reactions were routinely monitored by thin layer chromatography (TLC). All the reported melting points were taken in open capillaries and were uncorrected. Infrared (IR) spectra were recorded in KBr (pellet forms) on a Thermo Nicolet-Avatar-330 Fourier Transform Infrared (FT-IR) spectrophotometer and only noteworthy absorption values (cm^−1^) were listed. ^1^H and ^13^C NMR (nuclear magnetic resonance) were recorded with Bruker AMX-400 spectrometer at 400 and 100 MHz, respectively. NMR spectra were obtained in DMSO-*d*_6_ solutions and are reported as parts per million (ppm) downfield from a tetramethylsilane internal standard. Mass spectrometry is recorded with Applied Biosystems mass spectrometer. Elemental analyses (C, H, and N) were performed using the Thermo Scientific Flash 2000 organic elemental analyzer. Merck silica gel (100–200 mesh) was used for column chromatography.

#### 4.1.1. Typical Procedure for Synthesis of 2-Chloro-1-(1-aryl-1*H*-tetrazol-5-yl)ethanone (**15–21**)

A 100 mL RB flask was charged with 1-aryl-1*H*-tetrazole (1 mmol), chloroacetyl chloride (2.5 mmol) and pyridine (0.1 mmol) in tetrahydrofuran (25 mL) at 0°C. The reaction mixture was refluxed for 6 hrs. The flow of the reaction was monitored by TLC. After the completion of the reaction, the reaction mixture was quenched with crushed ice and the solid thrown out was filtered, washed with water, and dried under vacuum to obtain white solid. Finally, the crude product was purified through the column chromatography.

#### 4.1.2. General Procedure for Synthesis of 1-(1-Aryl-1*H*-tetrazol-5-yl)-2-(piperidin-1-yl)ethanone (**22–28**)

A 150 mL conical flask was charged with 2-chloro-1-(1-aryl-1*H*-tetrazol-5-yl)ethanone (1 mmol), piperidine (1.2 mmol), and triethylamine (0.1 mmol) in acetonitrile (25 mL). The reaction mixture was stirred for 6 hrs at room temperature. The reaction was monitored by TLC. After the completion of the reaction, the reaction mixture was quenched with crushed ice and the solid was filtered, washed with water, and dried under vacuum to get novel 2-(1*H*-imidazol-1-yl)-1-(1-aryl-1*H*-tetrazol-5-yl)ethanone. Finally, the crude product was purified through the column chromatography.

#### 4.1.3. 1-(1-Phenyl-1*H*-tetrazol-5-yl)-2-(piperidin-1-yl)ethanone (**22**)

IR (cm^−1^): 3428, 3314, 1656, 1592, 1551, 1448, 1357, 1255. ^1^H NMR (DMSO-*d*_*6*_, *δ* ppm): 1.37 (s, 2H, CH_2_), 1.51 (s, 4H, 2CH_2_), 1.51 (s, 4H, 2CH_2_), 3.15 (s, 2H, CH_2_), 7.07–7.10 (t, 1H, Ar-H), 7.31–7.34 (t, 2H, Ar-H), 7.50–7.52 (d, *J* = 8 Hz, 2H, Ar-H).^ 13^C NMR (DMSO-*d*_*6*_, *δ* ppm): 23.36, 25.48, 53.73, 61.44, 119.62, 123.72, 128.95, 137.19, 150.19, 172.79. MS,* m/z*: 271 (M+1). Elemental analysis found (calculated) for C_14_H_17_N_5_O (%): C, 61.89 (61.98); H, 6.26 (6.32); N, 25.61 (25.81).

#### 4.1.4. 2-(Piperidin-1-yl)-1-(1-p-tolyl-1*H*-tetrazol-5-yl)ethanone (**23**)

IR (cm^−1^): 3428, 3314, 1655, 1614, 1592, 1295. ^1^H NMR (DMSO-*d*_*6*_, *δ* ppm): 1.29–1.32 (m, 2H, CH_2_), 1.29–1.34 (m, 4H, 2CH_2_), 1.44–1.48 (m, 4H, 2CH_2_), 2.19 (s, 3H, CH_3_), 3.08 (s, 2H, CH_2_), 7.05–7.07 (d, *J* = 8 Hz, 2H, Ar-H), 7.32–7.34 (d, *J* = 8 Hz, 2H, Ar-H).^ 13^C NMR (DMSO-*d*_*6*_, *δ* ppm): 20.36, 23.36, 25.49, 53.74, 61.44, 119.63, 129.9, 132.71, 134.89, 150.12, 172.71. MS,* m/z*: 301 (M+1). Elemental analysis found (calculated) for C_15_H_19_N_5_O_2 _(%): C, 59.70 (59.79); H, 6.28 (6.36); N, 23.04 (23.24).

#### 4.1.5. 1-(1-(4-Methoxyphenyl)-1*H*-tetrazol-5-yl)-2-(piperidin-1-yl)ethanone (**24**)

IR (cm^−1^): 3470, 3298, 1655, 1586, 1557. ^1^H NMR (DMSO-*d*_*6*_, *δ* ppm): 1.38 (s, 2H, CH_2_), 1.52 (m, 4H, 2CH_2_), 2.46 (m, 4H, 2CH_2_), 3.14 (s, 2H, CH_2_), 3.72 (s, 3H, OCH_3_), 6.89–6.91 (d, *J* = 8 Hz, 2H, Ar-H), 7.41–7.43 (d, *J* = 8 Hz, 2H, Ar-H).^ 13^C NMR (DMSO-*d*_*6*_, *δ* ppm): 23.38, 25.51, 53.74, 55.15, 61.44, 114.14, 121.39, 130.38, 150.23, 156.63, 172.65. MS,* m/z*: 285 (M+1). Elemental analysis found (calculated) for C_15_H_19_N_5_O (%): C, 62.98 (63.14); H, 6.59 (6.71); N, 24.44 (24.54).

#### 4.1.6. 1-(1-(4-Chlorophenyl)-1*H*-tetrazol-5-yl)-2-(piperidin-1-yl)ethanone (**25**)

IR (cm^−1^): 3424, 3321, 1654, 1594, 1549. ^1^H NMR (DMSO-*d*_*6*_, *δ* ppm): 1.22 (s, 2H, CH_2_), 1.34 (s, 4H, 2CH_2_), 1.48 (s, 4H, 2CH_2_), 7.50 (s, 4H).^ 13^C NMR (DMSO-*d*_*6*_, *δ* ppm): 25.49, 53.74, 61.44, 119.63, 121.9, 129.39, 130.38, 150.23, 156.63, 172.65. MS,* m/z*: 305 (M+1). Elemental analysis found (calculated) for C_14_H_16_ClN_5_O (%): C, 54.80 (54.99); H, 5.19 (5.27); N, 22.75 (22.90).

#### 4.1.7. 1-(1-(4-Bromophenyl)-1*H*-tetrazol-5-yl)-2-(piperidin-1-yl)ethanone (**26**)

IR (cm^−1^): 3472, 3336, 1646, 1592, 1546. ^1^H NMR (DMSO-*d*_*6*_, *δ* ppm): 1.38 (s, 4H, 2CH_2_), 1.52 (s, 4H, 2CH_2_), 2.45 (s, 4H, 2CH_2_), 3.15 (s, 4H, 2CH_2_), 7.51 (s, 4H, Ar-H).^ 13^C NMR (DMSO-*d*_*6*_, *δ* ppm): 23.36, 25.49, 53.72, 61.45, 115.33, 121.65, 131.69, 136.93, 150.21, 172.72. MS,* m/z*: 349 (M+1). Elemental analysis found (calculated) for C_14_H_16_BrN_5_O (%): C, 48.15 (48.01); H, 4.45 (4.60); N, 19.79 (20.00).

#### 4.1.8. 1-(1-(4-Fluorophenyl)-1*H*-tetrazol-5-yl)-2-(piperidin-1-yl)ethanone (**27**)

IR (cm^−1^): 3314, 1656, 1592, 1551, 1448, 1357. ^1^H NMR (DMSO-*d*_*6*_, *δ* ppm): 1.26 (s, 2H, CH_2_), 1.38 (s, 4H, 2CH_2_), 1.52 (s, 4H, 2CH_2_), 7.08–7.10 (d, *J* = 8 Hz, 2H, Ar-H), 7.32–7.34 (d, *J* = 8 Hz, 2H, Ar-H).^ 13^C NMR (DMSO-*d*_*6*_, *δ* ppm): 25.56, 57.74, 65.40, 123.63, 122.9, 129.39, 136.28, 152.23, 156.63, 174.60. MS,* m/z*: 289 (M+1). Elemental analysis found (calculated) for C_14_H_16_FN_5_O (%): C, 57.98 (58.12); H, 5.48 (5.57); N, 23.98 (24.21).

#### 4.1.9. 1-(1-(4-Nitrophenyl)-1*H*-tetrazol-5-yl)-2-(piperidin-1-yl)ethanone (**28**)

IR (cm^−1^): 3448, 3364, 1655, 1618, 1582. ^1^H NMR (DMSO-*d*_*6*_, *δ* ppm): 1.38 (s, 2H, CH_2_), 1.52 (m, 4H, 2CH_2_), 2.46 (m, 4H, 2CH_2_), 3.14 (s, 2H, CH_2_), 6.89–6.91 (d, *J* = 8 Hz, 2H, Ar-H), 7.41–7.43 (d, *J* = 8 Hz, 2H, Ar-H).^ 13^C NMR (DMSO-*d*_*6*_, *δ* ppm): 23.38, 25.51, 53.74, 55.15, 62.49, 118.14, 121.39, 132.38, 153.23, 158.63, 170.65. MS,* m/z*: 316 (M+1). Elemental analysis found (calculated) for C_14_H_16_N_6_O_3_ (%): C, 48.15 (53.16); H, 4.96 (5.10); N, 26.45 (26.57).

## Figures and Tables

**Table 1:** Physical data for the newly synthesized compounds **22–28**.

Compounds	X	Yield (%)	Mp (°C)
**22**	H	82	165–168
**23**	CH_3_	79	145–148
**24**	OCH_3_	70	160–162
**25**	Cl	75	154–158
**26**	Br	68	180–182
**27**	F	78	172–176
**28**	NO_2_	60	170–172

**Table 2:** *In vitro* antibacterial activities of **22–28** against clinically isolated bacterial strains.

Compound	Minimum inhibitory concentration (MIC) in *μ*g/mL					
	*S. aureus *	*B. subtilis *	*S. Typhi*	*V. cholerae *	*E. coli *	*K. pneumoniae *

**22**	25	25	50	50	100	100
**23**	—	100	—	100	50	50
**24**	50	50	100	50	25	100
**25**	12.5	50	25	25	6.25	—
**26**	100	100	100	—	25	100
**27**	25	25	50	25	6.25	50
**28**	12.5	12.5	25	12.5	6.25	25
Ciprofloxacin	12.5	25	25	12.5	12.5	25

“—” indicates no inhibition even at a higher concentration of 200 *μ*g/mL.

**Table 3:** *In vitro* antifungal activities of **22–28** against clinically isolated fungal strains.

Compound	Minimum inhibitory concentration (MIC) in *μ*g/mL					
	*A. flavus *	*A. niger *	*C. albicans *	*Mucor *	*Candida 6 *	*Rhizopus *
**22**	50	50	50	50	50	50
**23**	—	100	25	100	100	100
**24**	50	50	50	—	50	50
**25**	12.5	50	25	25	25	—
**26**	50	100	50	50	50	100
**27**	50	25	6.25	25	12.5	50
**28**	25	25	12.5	12.5	25	50
Fluconazole	12.5	25	12.5	25	25	50

“—” indicates no inhibition even at a higher concentration of 200 *μ*g/mL.

**Table 4:** Molecular docking results of the target molecules with *multidrug  transporter EmrD* from *Escherichia coli* (PDB ID: **2GFP**).

Compound	Binding energy (kcal/mol)	Docking energy (kcal/mol)	Inhibition constant (*μ*M)	Intermolec. energy (kcal/mol)
**22**	−8.18	−9.38	10.0	−9.35
**23**	−8.28	−9.86	8.50	−9.34
**24**	−7.68	−7.86	4.30	−7.88
**25**	−9.57	−10.84	2.35	−10.87
**26**	−8.68	−9.86	9.33	−9.88
**27**	−8.18	−9.38	10.0	−9.22
**28**	−9.37	−10.72	13.6	−10.69

**Table 5:** Molecular docking results of the target molecules with *dihydrofolate reductase* from *Candidaalbicans* (PDB ID: **1AI9**).

Compound	Binding energy (kcal/mol)	Docking energy (kcal/mol)	Inhibition constant (*μ*M)	Intermolec. energy (kcal/mol)
**22**	−6.60	−7.54	14.47	−7.52
**23**	−7.11	−7.74	6.09	−7.79
**24**	−6.20	−7.59	28.74	−7.77
**25**	−7.59	−8.91	2.72	−8.90
**26**	−7.01	−7.70	7.25	−7.70
**27**	−7.53	−8.70	3.04	−8.68
**28**	−7.60	−9.27	2.63	−9.23
